# Coherence measure in terms of the Tsallis relative *α* entropy

**DOI:** 10.1038/s41598-017-18692-1

**Published:** 2018-01-10

**Authors:** Haiqing Zhao, Chang-shui Yu

**Affiliations:** 1 College of Science, Dalian jiaotong University, Dalian, 116028 China; 2 School of Physics, Dalian University of Technology, Dalian, 116024 China

## Abstract

Coherence is the most fundamental quantum feature of the nonclassical systems. The understanding of coherence within the resource theory has been attracting increasing interest among which the quantification of coherence is an essential ingredient. A satisfactory measure should meet certain standard criteria. It seems that the most crucial criterion should be the strong monotonicity, that is, average coherence doesn’t increase under the (sub-selective) incoherent operations. Recently, the Tsallis relative *α* entropy has been tried to quantify the coherence. But it was shown to violate the strong monotonicity, even though it can unambiguously distinguish the coherent and the incoherent states with the monotonicity. Here we establish a family of coherence quantifiers which are closely related to the Tsallis relative *α* entropy. It proves that this family of quantifiers satisfy all the standard criteria and particularly cover several typical coherence measures.

## Introduction

Coherence, the most fundamental quantum feature of a nonclassical system, stems from quantum superposition principle which reveals the wave particle duality of matter. It has been shown that coherence plays the key roles in the physical dynamics in biology^1–7^, transport theory^8,9^, and thermodynamics^10–14^. In particular, some typical approaches such as phase space distributions and higher order correlation functions have been developed in quantum optics to reveal quantum coherence even as an irrigorous quantification^15–17^. Quite recently, quantum coherence has been attracting increasing interest in various aspects^18–33^ including the quantification of coherence^18–23^, the operational resource theory^24–28^, the distribution^29^, the different understandings^34–41^ and so on.

Quantification of coherence is the most essential ingredient not only in the quantum theory but also in the practical application. Various quantities have been proposed to serve as a coherence quantifier, however the available candidates are still quite limited. Up to now, only two alternatives, i.e., the coherence measures based on *l*_1_ norm and the relative entropy, have turned out to be a satisfactory coherence measure^18^. In contrast, the usual *l*_*p*_ (*p* ≠ 1) norm can not directly induce a good measure^19^. In addition, the coherence quantifier based on the Fidelity is easily shown to satisfy the monotonicity that the coherence of the post-incoherent-operation state doesn’t increase, but it violates the strong monotonicity that average coherence doesn’t increase under the sub-selective incoherent operations^18,42^. Similarly, the coherence based on the trace norm is valid in many cases^19,42^ but looks invalid in general^43^. However, we know that the strong monotonicity is much more important than the monotonicity not only because the sub-selection of the measurement outcomes required by the strong monotonicity can be well controlled in experiment as is stated in refs^18,19^, but also because the realizable sub-selection would lead to the real increment of the coherence from the point of resource theory of view if the strong monotonicity was violated. In this sense, the quantitative characterization of coherence still needs to be paid more attention.

Recently, ref.^22^ has also proposed a coherence quantifier in terms of the Tsallis relative *α* entropy which lays the foundation to the non-extensive thermo-statistics and plays the same role as the standard logarithmic entropy does in the information theory^44,45^. However, it is unfortunate that the Tsallis relative *α* entropy isn’t an ideal coherence measure either because ref.^22^ showed that it only satisfies the monotonicity and a variational monotonicity rather than the strong monotonicity. Is it possible to bridge the Tsallis relative *α* entropy with the strong monotonicity by some particular and elaborate design? In this paper, we build such a bridge between the Tsallis relative *α* entropy with the strong monotonicity, hence present a family of good coherence quantifiers. By considering the special case in this family, one can find that the *l*_2_ norm can be validly employed to quantify the coherence. The remaining of this paper is organized as follows. We first introduce the coherence measure and the Tsallis relative *α* entropy. Then we present the family of coherence quantifier and mainly prove them to be strongly monotonic, and then we study the maximal coherence, several particular coherence measures and give a concrete application. Finally, we finish the paper by the conclusion and some discussions.

## Result

### The coherence and the Tsallis relative *α* entropy

The resource theory includes three ingredients: the free states, the resource states and the free operations^24,46^. For coherence, the free states are referred to as the incoherent states which are defined in a given fixed basis {|*i*〉} by the states with the density matrices in the diagonal form, i.e., $\delta ={\sum }_{i}{\delta }_{i}|i\rangle \langle i|$ with ${\sum }_{i}{\delta }_{i}=1$ for the positive *δ*_*i*_. All the states without the above diagonal form are the coherent states, i.e., the resource states. The quantum operations described by the Kraus operators {*K*_*n*_} with ${K}_{n}^{\dagger }{K}_{n}={\bf{I}}$ are called as the incoherent operations and serve as the free operations for coherence, if ${K}_{n}\delta {K}_{n}^{\dagger }\in  {\mathcal I}$ for any incoherent *δ*. In this sense, the standard criteria of a good coherence quantifier *C*(*ρ*) for the state *ρ* can be rigorously rewritten as^18^ (i) (Null) *C*(*δ*) = 0 for $\delta \in  {\mathcal I}$; (ii) (Strong monotonicity) for any state *ρ* and incoherent operations {*K*_*n*_}, $C(\rho ){\rm{\ge }}{\sum }_{n}\,{p}_{n}C({\rho }_{n})$ with ${p}_{n}={\rm{Tr}}{K}_{n}\rho {K}_{n}^{\dagger }$ and ${\rho }_{n}={K}_{n}\rho {K}_{n}^{\dagger }/{p}_{n}$; (iii) (Convexity) For any ensemble {*q*_*i*_, *σ*_*i*_}, $C({\sum }_{i}{q}_{i}{\sigma }_{i})\le {\sum }_{i}{q}_{i}C({\sigma }_{i})$.

In addition, ref.^18^ also introduces the monotonicity (in contrast to the *strong* monotonicity) that requires $C(\rho )\ge C({\sum }_{n}{p}_{n}{\rho }_{n})$. This actually can be automatically implied by (ii) and (iii). As mentioned in ref.^18^, the monotonicity is not laid in an important position compared with the strong monotonicity, because the measurement outcomes of {*K*_*n*_} can be well controlled (sub-selected) in practical experiments. In fact, the fundamental spirit of both the monotonicity and the strong monotonicity (or the resource theory) is to restrict that the coherence (resource) shouldn’t be increased under the incoherent (free) operations, which is parallel with the resource theory of entanglement, namely, the average entanglement is not increased under the local operations and classical communication (LOCC). However, if for a quantum state *ρ*, there exists one incoherent operation {*K*_*n*_} such that $C({\sum }_{n}{p}_{n}{\rho }_{n}) < C(\rho )$ but ${\sum }_{n}\,{p}_{n}C({\rho }_{n}) > C(\rho )$ where *n* denotes the measurement outcome with the probability ${p}_{n}={\rm{Tr}}{K}_{n}\rho {K}_{n}^{\dagger }$, and the corresponding post-measurement state is ${\rho }_{n}={K}_{n}\rho {K}_{n}^{\dagger }$, this means that if we erase the information of the measurement outcomes, the coherence of the post-measurement state *ρ*′ is less than the coherence of the pre-measurement state, but if we keep the measurement information, the average coherence is increased. However, in the practical experiment, it is not necessary for us to erase any information. This means that the incoherent operation {*K*_*n*_} can increase the coherence, which violates the fundamental spirit of a resource theory. It is why we emphasize the strong monotonicity.

With the above criteria, any measure of distinguishability such as the (pseudo-) distance norm could induce a potential candidate for a coherence quantifier. But it has been shown that some candidates only satisfy the monotonicity rather than the strong monotonicity, so they are not ideal and could be only used in the limited cases. ref.^22^ found that the coherence based on the Tsallis relative *α* entropy is also such a coherence quantifier without the strong monotonicity.

The Tsallis relative *α* entropy is a special case of the quantum *f*-divergences^22,47^. For two density matrices *ρ* and *σ*, it is defined as

1 $${D}_{\alpha }(\rho \parallel \sigma )=\frac{1}{\alpha -1}({\rm{Tr}}{\rho }^{\alpha }{\sigma }^{1-\alpha }-1)$$

for *α* ∈ (0, 2]. It is shown that for *α* → 1, ${D}_{\alpha }(\rho \parallel \sigma )$ will reduce to the relative entropy $S(\rho \parallel \sigma )=Tr\rho \,{\mathrm{log}}_{2}\,\rho -\rho \,{\mathrm{log}}_{2}\,\sigma$. The Tsallis relative *α* entropy ${D}_{\alpha }(\rho \parallel \sigma )$ inherits many important properties of the quantum *f*-divergences, for example, (Positivity) ${D}_{\alpha }(\rho \parallel \sigma )\ge 0$ with equality if and only if *ρ* = *σ*, (Isometry) ${D}_{\alpha }(U\rho {U}^{\dagger }\parallel U\sigma {U}^{\dagger })={D}_{\alpha }(\rho \parallel \sigma )$ for any unitary operations, (Contractibility) ${D}_{\alpha }(\$(\rho )\parallel \$(\sigma ))\le {D}_{\alpha }(\rho \parallel \sigma )$ under any trace-preserving and completely positive (TPCP) map $\$$ and (Joint convexity) ${D}_{\alpha }({\sum }_{n}\,{p}_{n}{\rho }_{n}\parallel {\sum }_{n}\,{p}_{n}{\sigma }_{n})\le {\sum }_{n}\,{p}_{n}{D}_{\alpha }({\rho }_{n}\parallel {\sigma }_{n})$ for the density matrices *ρ*_*n*_ and *σ*_*n*_ and the corresponding probability distribution *p*_*n*_.

Based on the Tsallis relative *α* entropy ${D}_{\alpha }(\rho \parallel \sigma )$, the coherence in the fixed reference basis {|*i*〉} can be characterized by^22^

2 $${\tilde{C}}_{\alpha }(\rho )=\mathop{{\rm{\min }}}\limits_{\delta \in  {\mathcal I} }\,{D}_{\alpha }(\rho \parallel \delta )=\frac{1}{\alpha -1}[{(\sum _{j}\langle j|{\rho }^{\alpha }{|j\rangle }^{\mathrm{1/}\alpha })}^{\alpha }-1]\mathrm{.}$$

However, it is shown that ${\tilde{C}}_{\alpha }(\rho )$ satisfies all the criteria for a good coherence measure but the strong monotonicity. Since ${D}_{\alpha \to 1}(\rho \parallel \sigma )$ reduces to the relative entropy $S(\rho \parallel \sigma )$ which has induced the good coherence measure, throughout the paper we are mainly interested in $\alpha \in \mathrm{(0},\mathrm{1)}\cup \mathrm{(1},\mathrm{2]}$.

In addition, the Tsallis relative *α* entropy ${D}_{\alpha }(\rho \parallel \sigma )$ can also be reformulated by a very useful function as

3 $${D}_{\alpha }(\rho \parallel \sigma )=\frac{1}{\alpha -1}({f}_{\alpha }(\rho ,\sigma )-1)$$

with

4 $${f}_{\alpha }(\rho ,\sigma )={\rm{Tr}}{\rho }^{\alpha }{\sigma }^{1-\alpha }\mathrm{.}$$

Accordingly, the coherence ${\tilde{C}}_{\alpha }(\rho )$ can also be rewritten as

5 $${\tilde{C}}_{\alpha }(\rho )=\frac{1}{\alpha -1}[{{\rm{sgn}}}_{1}(\alpha )\,\mathop{{\rm{\min }}}\limits_{\delta \in  {\mathcal I} }\,{{\rm{sgn}}}_{1}(\alpha ){f}_{\alpha }(\rho ,\delta )-1]$$

which, based on Eq. (2), leads to the conclusion

6 $$\mathop{{\rm{\min }}}\limits_{\delta \in  {\mathcal I} }\,{{\rm{sgn}}}_{1}(\alpha ){f}_{\alpha }(\rho ,\delta )={(\sum _{j}\langle j|{\rho }^{\alpha }{|j\rangle }^{\mathrm{1/}\alpha })}^{\alpha }\mathrm{.}$$

Based on Eq. (6) and the properties of ${D}_{\alpha }(\rho \parallel \sigma )$ mentioned above, one can have the following observations for the function *f*_*α*_(*ρ*, *σ*)^22,47^.

**Observations:** *f*_*α*_(*ρ*, *σ*) satisfies the following properties:

(I) *f*_*α*_(*ρ*, *σ*) ≥ 1 for *α* ∈ (1, 2] and *f*_*α*_(*ρ*, *σ*) ≤ 1 for *α* ∈ (0, 1) with equality if and only if *ρ* = *σ*;
(II) For a unitary operation *U*, *f*_*α*_(*UρU* ^†^, *UσU* ^†^) = *f*_*α*_(*ρ*, *σ*);
(III) For any TPCP map $, *f*_*α*_(*ρ*, *σ*) doesn’t decrease for *α* ∈ (0, 1), and doesn’t increased for *α* ∈ (1, 2], namely,
  7 $${{\rm{sgn}}}_{1}(\alpha ){f}_{\alpha }(\$[\rho ],\$[\sigma ])\le {{\rm{sgn}}}_{1}(\alpha ){f}_{\alpha }(\rho ,\sigma ),$$
  where the function is defined by $${{\rm{sgn}}}_{1}(\alpha )=\{\begin{array}{cc}-\mathrm{1,} & \alpha \in \mathrm{(0,1)}\\ \mathrm{1,} & \alpha \in \mathrm{(1,2]}\end{array}$$;
(IV) The function sgn_1_(*α*)*f*_*α*_(*ρ*, *σ*) is jointly convex;
(V) For a state *δ*, $${f}_{\alpha }(\rho \otimes \delta ,\sigma \otimes \delta )={f}_{\alpha }(\rho \parallel \sigma )$$, which can be easily found from the function itself.

### The coherence measures based on the Tsallis relative *α* entropy

To proceed, we would like to present a very important lemma for the function *f*_*α*_(*ρ*, *σ*), which is the key to show our main result.

#### **Lemma 1**

Suppose both *ρ* and *σ* simultaneously undergo a TPCP map $\$\,:=\{{M}_{n}:\sum _{n}\,{M}_{n}^{\dagger }{M}_{n}={{\mathbb{I}}}_{S}\}$ which transforms the states *ρ* and *σ* into the ensemble {*p*_*n*_, *ρ*_*n*_} and {*q*_*n*_, *σ*_*n*_}, respectively, then we have

8 $${{\rm{sgn}}}_{1}(\alpha ){f}_{\alpha }({\rho }_{S},{\delta }_{S})\ge {{\rm{sgn}}}_{1}(\alpha )\,\sum _{n}\,{p}_{n}^{\alpha }{q}_{n}^{1-\alpha }{f}_{\alpha }({\rho }_{n},{\sigma }_{n})\mathrm{.}$$

The proof is given in the **Methods**.

Based on Lemma 1 and the preliminaries given in the previous section, we can present our main theorem as follows.

#### **Theorem 1**

The coherence of a quantum state *ρ* can be measured by

9 $$\begin{array}{rcl}{C}_{\alpha }(\rho ) & = & \mathop{{\rm{\min }}}\limits_{\delta \in  {\mathcal I} }\frac{1}{\alpha -1}({f}_{\alpha }^{\mathrm{1/}\alpha }(\rho ,\delta )-1)\end{array}$$

10 $$\begin{array}{rcl} & = & \frac{1}{\alpha -1}(\sum _{j}\langle j|{\rho }^{\alpha }{|j\rangle }^{\mathrm{1/}\alpha }-1),\end{array}$$

where *α* ∈ (0, 2], {|*j*〉} is the reference basis and ${f}_{\alpha }(\rho ,\delta )=(\alpha -1){D}_{\alpha }(\rho \parallel \sigma )+1$ with ${D}_{\alpha }(\rho \parallel \sigma )$ representing the Tsallis relative *α* entropy.

#### **Proof**

. At first, one can note that the function *x* ^*α*^ is a monotonically increasing function on *x*, so Eq. (10) obviously holds for positive *x* due to Eq. (6).

#### Null

Since the original Tsallis entropy defined by Eq. (2) can unambiguously distinguish a coherent state from the incoherent one. Eq. (2) implies that ${\sum }_{j}\,\langle j|{\rho }^{\alpha }{|j\rangle }^{\mathrm{1/}\alpha }=1$ is sufficient and necessary condition for incoherent states. Thus the zero *C*_*α*_(*ρ*) is also a sufficient and necessary condition for incoherent state *ρ*.

#### Convexity

From ref.^48^, one can learn that the function *g*(*A*) = Tr(*XA* ^*p*^ *X* ^†^)^*s*^ is convex in positive matrix *A* for *p* ∈ [1, 2] and $s\ge \frac{1}{p}$, and concave in *A* for *p* ∈ (0, 1] and $1\le s\le \frac{1}{p}$. Now let’s assume *A* = *ρ*, $X=|j\rangle \langle j|$ and *p* = *α* and $s=\frac{1}{\alpha }$, thus one has

11 $${g}_{\alpha }^{j}(\rho )={\rm{Tr}}{(|j\rangle \langle j|{\rho }^{\alpha }|j\rangle \langle j|)}^{\mathrm{1/}\alpha }=\langle j|{\rho }^{\alpha }{|j\rangle }^{\mathrm{1/}\alpha },$$

which implies ${g}_{\alpha }^{j}(\rho )$ is convex in density matrix *ρ* for *α* ∈ [1, 2] and $s=\frac{1}{\alpha }$, and concave in *ρ* for *α* ∈ (0, 1] and $s=\frac{1}{\alpha }$. Here the subscript *α* and the superscript *j* in ${g}_{\alpha }^{j}$ specifies the particular choice. So it is easy to find that $\frac{1}{\alpha -1}{\sum }_{j}\,{g}_{\alpha }^{j}(\rho )$ is convex for *α* ∈ (0, 2]. Considering Eq. (10), one can easily show *C*_*α*_(*ρ*) is convex in *ρ*.

#### Strong monotonicity

Now let {*M*_*n*_} denote the incoherent operation, so the ensemble after the incoherent operation on the state *ρ* can be given by {*p*_*n*_,*ρ*_*n*_} with ${p}_{n}={\rm{Tr}}{M}_{n}\rho {M}_{n}^{\dagger }$ and ${\rho }_{n}={M}_{n}\rho {M}_{n}^{\dagger }/{p}_{n}$. Thus the average coherence ${\bar{C}}_{\alpha }$ is

12 $${\bar{C}}_{\alpha }=\sum _{n}\,{p}_{n}{C}_{\alpha }({\rho }_{n})=\mathop{{\rm{\min }}}\limits_{{\delta }_{n}\in  {\mathcal I} }\frac{1}{\alpha -1}(\sum _{n}\,{p}_{n}{f}_{\alpha }^{\mathrm{1/}\alpha }({\rho }_{n},{\delta }_{n})-1)\mathrm{.}$$

Let *δ* ^*o*^ denote the optimal incoherent state such that

13 $${C}_{\alpha }(\rho )=\frac{1}{\alpha -1}({f}_{\alpha }^{\mathrm{1/}\alpha }(\rho ,{\delta }^{o})-1),$$

i.e.,

14 $${f}_{\alpha }(\rho ,{\delta }^{o})=\mathop{{\rm{\min }}}\limits_{\delta \in  {\mathcal I} }\,{{\rm{sgn}}}_{1}(\alpha ){f}_{\alpha }(\rho ,\delta \mathrm{).}$$

Considering the incoherent operation {*M*_*n*_}, we have ${\sigma }_{n}^{o}={M}_{n}{\delta }^{o}{M}_{n}^{\dagger }/{q}_{n}\in  {\mathcal I}$ with ${q}_{n}={\rm{Tr}}{M}_{n}{\delta }^{o}{M}_{n}^{\dagger }$. Therefore, one can immediately find that

15 $$\mathop{{\rm{\min }}}\limits_{\delta \in  {\mathcal I} }\,{{\rm{sgn}}}_{1}(\alpha ){f}_{\alpha }^{\mathrm{1/}\alpha }(\rho ,\delta )\le {{\rm{sgn}}}_{1}(\alpha ){f}_{\alpha }^{\mathrm{1/}\alpha }({\rho }_{n},{\sigma }_{n}^{o}),$$

where we use the function *x* ^1/*α*^ is monotonically increasing on *x*. According to Eqs (12) and (15), we obtain

16 $${\bar{C}}_{\alpha }\le \frac{1}{\alpha -1}(\sum _{n}\,{p}_{n}\,{f}_{\alpha }^{\mathrm{1/}\alpha }({\rho }_{n},{\sigma }_{n}^{o})-1)\mathrm{.}$$

In addition, the Hölder inequality^49^ implies that for *α* ∈ (0, 1),

17 $${[\sum _{n}{q}_{n}]}^{1-\alpha }\,{[\sum _{n}{p}_{n}{f}_{\alpha }^{\mathrm{1/}\alpha }({\rho }_{n},{\sigma }_{n}^{o})]}^{\alpha }\ge \sum _{n}\,{p}_{n}^{\alpha }{q}_{n}^{1-\alpha }{f}_{\alpha }({\rho }_{n},{\sigma }_{n}^{o}),$$

and the inequality sign is reverse for *α* ∈ (1, 2], so Eq. (16) becomes

18 $${\bar{C}}_{\alpha }\le \frac{1}{\alpha -1}({[\sum _{n}{p}_{n}^{\alpha }{q}_{n}^{1-\alpha }{f}_{\alpha }({\rho }_{n},{\sigma }_{n}^{o})]}^{\mathrm{1/}\alpha }-1)\le \frac{1}{\alpha -1}({f}_{\alpha }^{\mathrm{1/}\alpha }(\rho ,{\delta }^{o})-1)={C}_{\alpha },$$

which is due to Lemma 1. Eq. (18) shows the strong monotonicity of *C*_*α*_.               ■

### Maximal coherence and several typical quantifiers

Next, we will show that the maximal coherence can be achieved by the maximally coherent states. At first, we assume *α* ∈ (0, 1). Based on the eigen-decomposition of a *d*-dimensional state $\rho :\rho ={\sum }_{k}{\lambda }_{k}|{\psi }_{k}\rangle \langle {\psi }_{k}|$ with *λ*_*k*_ and $|{\psi }_{k}\rangle$ representing the eigenvalue and eigenvectors, we have

19 $$\sum _{j}\,\langle j|{\rho }^{\alpha }{|j\rangle }^{\mathrm{1/}\alpha }=\sum _{j}\,{(\sum _{k}{\lambda }_{k}^{\alpha }{|\langle {\psi }_{k}|j\rangle |}^{2})}^{\mathrm{1/}\alpha }\ge d{(\sum _{jk}\frac{{\lambda }_{k}^{\alpha }}{d}{|\langle {\psi }_{k}|j\rangle |}^{2})}^{\mathrm{1/}\alpha }\ge d{(\sum _{k}\frac{{\lambda }_{k}^{\alpha }}{d})}^{\mathrm{1/}\alpha }\ge {d}^{\frac{\alpha -1}{\alpha }}.$$

One can easily find that the lower bound Eq. (19) can be attained by the maximally coherent states ${\rho }_{m}=|{\rm{\Psi }}\rangle \langle {\rm{\Psi }}|$ with $|{\rm{\Psi }}\rangle =\frac{1}{\sqrt{d}}\,{\sum }_{j}\,{e}^{i{\varphi }_{j}}|j\rangle$. Correspondingly, the coherence is given by ${C}_{0 < \alpha  < 1}({\rho }_{m})=\frac{1}{1-\alpha }(1-{d}^{\frac{\alpha -1}{\alpha }})$. Similarly, for *α* ∈ (1, 2], the function *x* ^1/*α*^ is concave, which leads to that Eq. (19) with the inverse inequality sign holds. The inequality can also saturate for *ρ*_*m*_. The corresponding coherence can be found to have the same form as *C*_0<*α*<1_(*ρ*_*m*_). In other words,

20 $${C}_{0 < \alpha  < 2}({\rho }_{m})=\frac{1}{1-\alpha }(1-{d}^{\frac{\alpha -1}{\alpha }})\mathrm{.}$$

*C*_*α*_(*ρ*) actually defines a family of coherence measures related to the Tsallis relative *α* entropy. This family includes several typical coherence measures. As mentioned above, the most prominent coherence measure belonging to this family is the coherence in terms of relative entropy, i.e., *C*_1_(*ρ*) = *S*(*ρ*).

One can also find that

21 $${C}_{\mathrm{1/2}}(\rho )=\mathop{{\rm{\min }}}\limits_{\delta \in  {\mathcal I} }\,2(1-{[Tr\sqrt{\rho }\sqrt{\delta }]}^{2})=\mathop{{\rm{\min }}}\limits_{\delta \in  {\mathcal I} }\,{\Vert \sqrt{\rho }-\sqrt{\delta }\Vert }_{2}^{2}=1-\sum _{i}\,\langle i|\sqrt{\rho }{|i\rangle }^{2}$$

with ||·||_2_ denoting *l*_2_ norm. So the *l*_2_ norm has been revived for coherence measure by considering the square root of the density matrices. This is much like the quantification of quantum correlation proposed in ref.^50^. In addition, *C*_1/2_(*ρ*) can also be rewritten as

22 $${C}_{\mathrm{1/2}}(\rho )=-\frac{1}{2}\,\sum _{i}\,{\rm{Tr}}\{{[\sqrt{\rho },|i\rangle \langle i|]}^{2}\}$$

which is just the coherence measure based on the skew information^51–53^.

Finally, one can also see that

23 $${C}_{2}(\rho )=\mathop{{\rm{\min }}}\limits_{\delta \in  {\mathcal I} }\,(\sqrt{Tr{\rho }^{2}{\delta }^{-1}}-1)=\sum _{i}\,\langle i|{\rho }^{2}{|i\rangle }^{\mathrm{1/2}}-1$$

which is a simple function of the density matrix.

### Applications

As applications, we would like to compare our coherence measure with other analytic coherence measures, that is, the measure based on *l*_1_ norm, the relative entropy and the skew information. Let’s consider a decoherence process where a bipartite maximally entangled state $$|\psi \rangle =\frac{1}{\sqrt{2}}(|++\rangle +|--\rangle )$$ with $$|\pm \rangle =\frac{1}{\sqrt{2}}(|0\rangle \pm |1\rangle )$$ undergoes a composite amplitude damping channel^54^ $${\rm{\$}}\otimes {\rm{\$}}$$ where $ = {*M*_*i*_} and $${M}_{1}=(\begin{array}{cc}1 & 0\\ 0 & \sqrt{1-\gamma }\end{array})$$, $${M}_{2}=(\begin{array}{cc}0 & \sqrt{\gamma }\\ 0 & 0\end{array})$$ with *γ* denoting the damping rate. Thus the final state under this amplitude damping channel can be given by

24 $$\begin{array}{rcl}\rho (\gamma ) & = & \$\otimes \$[|\psi \rangle \langle \psi |]\\  & = & \sum _{ij}\,{M}_{i}\otimes {M}_{j}|\psi \rangle \langle \psi |{M}_{i}^{\dagger }\otimes {M}_{j}^{\dagger }\\  & = & \frac{1}{4}(\begin{array}{cccc}{\mathrm{(1}+\gamma )}^{2} & 0 & 0 & 1-\gamma \\ 0 & 1-{\gamma }^{2} & 1-\gamma  & 0\\ 0 & 1-\gamma  & 1-{\gamma }^{2} & 0\\ 1-\gamma  & 0 & 0 & {\mathrm{(1}-\gamma )}^{2}\end{array})\mathrm{.}\end{array}$$

**Figure 1:**
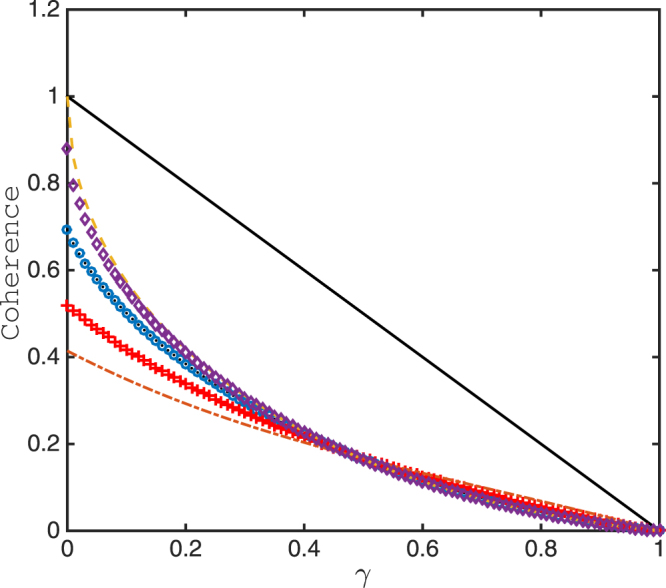
Coherence based on various measures versus *γ*. The solid line corresponds to ${C}_{{l}_{1}}$ and the dashed line corresponds to *C*_1/2_ which corresponds to the coherence in terms of skew information. The ‘diamond’ line, the ‘+’ line and the dash-dotted line, respectively correspond to *C*_2/3_, *C*_3/2_ and *C*_2_. In particular, the line marked by ‘o’ corresponds to *C*_*α*→1_ and the dot line corresponds to the coherence based on relative entropy *R*(*ρ*(*γ*)), which shows the perfect consistency.

Thus one can easily find that the coherence based on the *l*_1_ norm can be given by ${C}_{{l}_{1}}(\rho (\gamma ))={\sum }_{i\ne j}\,|{\rho }_{ij}|=1-\gamma$, and the coherence based on our Tsallis relative *α* entropy can be given by ${C}_{\alpha }(\rho (\gamma ))=\frac{1}{\alpha -1}({\sum }_{i=1}^{4}\,\langle i|\rho {(\gamma )}^{\alpha }{|i\rangle }^{\mathrm{1/}\alpha }-1)$. In particular, it is shown that *C*_*α*_(*ρ*(*γ*)) for *α* → 1 corresponds to the coherence based on the relative entropy defined by *R*(*ρ*(*γ*)) = *S*(I $\circ$ *ρ*(*γ*)) − *S*(*ρ*(*γ*)) with $\circ$ meaning the Hadamard product of matrices and *C*_1/2_(*ρ*(*γ*)) corresponds to the skew information^53^. In order to explicitly show the difference between the various coherence measures, we plot the coherence of the state *ρ*(*γ*) for ${C}_{{l}_{1}}$ and *C*_*α*_(*ρ*(*γ*)) for various *α* in Fig. 1.

## Conclusion

We establish a family of coherence measures that are closely related to the Tsallis relative *α* entropy. We prove that these coherence measures satisfy all the required criteria for a satisfactory coherence measure especially including the strong monotonicity. We also show this family of coherence measures includes several typical coherence measures such as the coherences measure based on von Neumann entropy, skew information and so on. Additionally, we show how to validate the *l*_2_ norm as a coherence measure. In addition, one can find that our current coherence measure can be easily related to the original Tsallis relative *α* entropy in Theorem 1, thus our current coherence measure has many potential applications or connections in both thermo-statistics and the information theory, since the Tsallis relative *α* entropy lays the foundation to the non-extensive thermo-statistics and have important applications in the information theory^44,45^. This could require the further investigation. Finally, we would like to emphasize that the convexity and the strong monotonicity could be two key points which couldn’t easily be compatible with each other to some extent. Fortunately, ref.^48^ provides the important knowledge to harmonize both points in this paper. This work builds the bridge between the Tsallis relative *α* entropy and the strong monotonicity and provides the important alternative quantifiers for the coherence quantification. This could shed new light on the strong monotonicity of other candidates for coherence measure.

## Methods

### **Proof of Lemma 1**

Any TPCP map can be realized by a unitary operation on a composite system followed by a local projective measurement^54^. Suppose system S is of our interest and A is an auxiliary system. For a TPCP map $\$\,:=\{{M}_{n}:{\sum }_{n}{M}_{n}^{\dagger }{M}_{n}={{\mathbb{I}}}_{S}\}$, one can always find a unitary operation *U*_*SA*_ and a group of projectors ${\{{{\rm{\Pi }}}_{n}^{A}=|n\rangle }_{A}\langle n|\}$ such that

25 $${M}_{n}{\rho }_{S}{M}_{n}^{\dagger }\otimes {{\rm{\Pi }}}_{n}^{A}=({{\mathbb{I}}}_{S}\otimes {{\rm{\Pi }}}_{n}^{A})\,{U}_{SA}({\rho }_{S}\otimes {{\rm{\Pi }}}_{0}^{A})\,{U}_{SA}^{\dagger }({{\mathbb{I}}}_{S}\otimes {{\rm{\Pi }}}_{n}^{A})\mathrm{.}$$

Using Properties (I) and (II), we have

26 $${f}_{\alpha }({\rho }_{S},{\delta }_{S})={f}_{\alpha }({U}_{SA}({\rho }_{S}\otimes {{\rm{\Pi }}}_{0}^{A})\,{U}_{SA}^{\dagger },{U}_{SA}({\sigma }_{S}\otimes {{\rm{\Pi }}}_{0}^{A})\,{U}_{SA}^{\dagger })$$

holds for any two states *ρ*_*S*_ and *σ*_*S*_. Let $${\rho }_{Sf}={\$}_{SA}[{U}_{SA}({\rho }_{S}\otimes {{\rm{\Pi }}}_{0}^{A}){U}_{SA}^{\dagger }]$$ and $${\sigma }_{Sf}={\$}_{SA}[{U}_{SA}({\sigma }_{S}\otimes {{\rm{\Pi }}}_{0}^{A}){U}_{SA}^{\dagger }]$$ which describe the states $${U}_{SA}({\rho }_{S}\otimes {{\rm{\Pi }}}_{0}^{A}){U}_{SA}^{\dagger }$$ and $${U}_{SA}({\sigma }_{S}\otimes {{\rm{\Pi }}}_{0}^{A}){U}_{SA}^{\dagger }$$ undergo an arbitrary TPCP map $_*SA*_ performed on the composite system S plus A. Based on Property (III), one can easily find

27 $${{\rm{sgn}}}_{1}(\alpha ){f}_{\alpha }({\rho }_{S},{\delta }_{S})\ge {{\rm{sgn}}}_{1}(\alpha ){f}_{\alpha }({\rho }_{Sf},{\sigma }_{Sf})\mathrm{.}$$

Suppose the TPCP map ${\$}_{SA}:=\{{{\mathbb{I}}}_{S}\otimes {{\rm{\Pi }}}_{n}^{A}\}$, according to Eq. (25), one can replace *ρ*_*Sf*_ and *σ*_*Sf*_ in Eq. (27), respectively, by

28 $${\rho }_{Sf}\to {\tilde{\rho }}_{Sf}=\sum _{n}\,{M}_{n}{\rho }_{S}\,{M}_{n}^{\dagger }\otimes {{\rm{\Pi }}}_{n}^{A}$$

and

29 $${\sigma }_{Sf}\to {\tilde{\sigma }}_{Sf}=\sum _{n}\,{M}_{n}{\sigma }_{S}{M}_{n}^{\dagger }\otimes {{\rm{\Pi }}}_{n}^{A}\mathrm{.}$$

Therefore, we get

30 $$\begin{array}{lcl}{{\rm{sgn}}}_{1}(\alpha ){f}_{\alpha }({\rho }_{S},{\delta }_{S}) & \ge  & {{\rm{sgn}}}_{1}(\alpha ){f}_{\alpha }({\tilde{\rho }}_{Sf},{\tilde{\sigma }}_{Sf})\\  & = & {{\rm{sgn}}}_{1}(\alpha )\,\sum _{n}\,{f}_{\alpha }({M}_{n}{\rho }_{S}{M}_{n}^{\dagger }\otimes {{\rm{\Pi }}}_{n}^{A},{M}_{n}{\sigma }_{S}{M}_{n}^{\dagger }\otimes {{\rm{\Pi }}}_{n}^{A})\\  & = & {{\rm{sgn}}}_{1}(\alpha )\,\sum _{n}\,{f}_{\alpha }({M}_{n}{\rho }_{S}{M}_{n}^{\dagger },{M}_{n}{\sigma }_{S}{M}_{n}^{\dagger })\\  & = & {{\rm{sgn}}}_{1}(\alpha )\,\sum _{n}\,{p}_{n}^{\alpha }{q}_{n}^{1-\alpha }{f}_{\alpha }({\rho }_{n},{\sigma }_{n}),\end{array}$$

which completes the proof                                 ■.
